# Indian traditional medicinal plants in ophthalmic diseases

**DOI:** 10.22038/AJP.2022.20345

**Published:** 2022

**Authors:** Sana Nafees, Jamal Akhtar, Jasbir Kaur

**Affiliations:** 1 *Department of Ocular Biochemistry, Dr. Rajendra Prasad Centre for Ophthalmic Sciences, All India Institute of Medical Sciences, New Delhi, India*; 2 *Central Council for Research in Unani Medicine, Ministry of AYUSH, Govt. of India, New Delhi, India *

**Keywords:** Ophthalmology, Atropa belladonna, Crocus sativus, Coptis teeta, Foeniculum vulgare, Gingko biloba

## Abstract

**Objective:**

Traditional herbal plants have been in use since ancient times to treat ophthalmic conditions; so, the aim of this study is to evaluate some potent Indian traditional medicinal plants used in ophthalmic diseases in order to summarize their potential effect in ophthalmology along with their mechanism of action.

**Materials and Methods:**

Databases PubMed, Google Scholar, and Embase were extensively explored. Additionally, relevant textbooks and literatures were consulted to summarize most of the considerable scientific literature for the review. Search term included ophthalmology, glaucoma, cataract, trachoma, conjunctivitis, traditional medicines, Unani drugs, and ayurvedic drugs were used. Around 80 review articles were consulted from the year 1982 to 2021.

**Results:**

The traditional medicinal plants are easily available, cost-effective and have no associated side effects in comparison to current conventional treatments. Moreover, these drugs in oppose to modern medicine, have an inherent potential to accelerate the body’s own immunity to fight against any infection. A large volume of scientific studies has reported the beneficial effects of traditional drugs in ophthalmology.

**Conclusion:**

This review, therefore, describes the potential benefits and uses of some traditional medicinal plants used in ophthalmic diseases.

## Introduction

The eye is the most important sensory organ of the body and it is extremely complex. Ophthalmic diseases afflict a substantial portion of the population and from ancient times humans have sought natural resources in the form of flora and fauna to treat eye diseases. WHO estimates that 80% of the world population rely on medicines of plant origin for their primary healthcare (WHO, 2019 ▶). Approximately there are 200 plants distributed worldwide that have been advocated in the treatment of eye disorders and numerous plant species have been documented in Traditional Indian Medicine for their ophthalmic effects (Pooja, 2014 ▶). 

Nature has been an unlimited source of biologically-active compounds. Attempts have been made to investigate new compounds with an aim to provide substantial benefits to eye tissue and vision with less side effects and toxicity (Newman and Cragg, 2016 ▶). Emerging scientific evidence of antimicrobial, antibacterial, antioxidant, anti-inflammatory, wound-healing, antitumor, and antiangiogenic actions of traditional medicines have encouraged more research investment in this area (Pinheiro, 2018 ▶). The search for a drug that effectively lowers intraocular pressure, thereby controlling the progression of glaucoma, has led to the development of various ocular hypotensive agents, such as physostigmine from *Physostigma venenosum* Balf. 

Discovery of plant-based antiglaucoma drugs and anaesthetizing agent for ocular tissue has led to the exponential growth in ocular pharmaceutics (Ruetsch, 2001 ▶). Despite technological advances in synthesizing drugs, the pharmaceutical industry still seeks new active compounds from natural sources as well as from revisiting already-established naturally derived compounds. However, the emergence of safer and more effective ophthalmic drugs has been depending on the scientific studies in the various areas of ophthalmology. 

The traditional herbal medicines have potential to overcome the limitations associated with conventional drugs. Therefore, many efforts have been made to identify new medicinal plants from different sources because of their effectiveness, particularly because they are locally available and easily consumable as raw or in simple medicinal preparations. Demand of the herbal drugs is becoming universal as the people are becoming more and more cognisant about the benefits of medicines with plant origin (Srikanth, 2014 ▶). Herbal drugs being easily affordable are becoming the first preference for the common man over purchasing the expensive modern pharmaceuticals. 

In spite of tremendous technological interventions in ophthalmic medicine and surgery, conservative treatments still continue to be mainstay for reversible ailments. Scientists and researchers are relentlessly in quest to identify plants with medicinal properties in maintaining the prolonged and healthy vision. However, despite incredible research and utilization of technological advancements, problems retained with management of conditions such as retinitis pigmentosa, chronic allergic disorders of adnexa, degenerative -neuro-ophthalmic lesions, etc. Apart from these problems, adverse effects of synthetic medications are generating a significant amount of discomfort and morbidity to the patients (Srikanth, 2007 ▶). This has become a challenge to the ophthalmologist, which requires special attention to develop and explore the hidden medical knowledge for better management of ophthalmic conditions. This review article has tried to submerge the effect of some traditional medicinal plants that are extensively investigated in various ophthalmic diseases and can further be evaluated for other ophthalmic diseases. 

***Atropa belladonna*** **L.**

**Figure 1:**
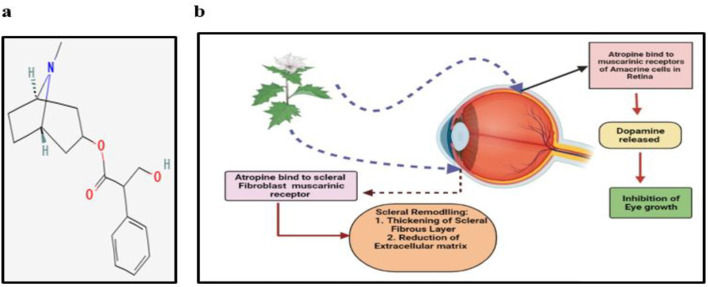
a: Chemical structure of Atropine. b: Mechanism of action of Atropine in myopia

The topical use of the macerated fruit of *Atropa belladonna *L. by the Egyptians is the first known use of a nature-derived agent to treat an ophthalmic disease (Duncan and Collison, 2003 ▶). The main chemical constituent of this plant is atropine, chemical structure is shown in Figure 1a. Chia et al. investigated in a randomized clinical trial that atropine at a low concentration (0.01% eyedrops) was more effective in slowing myopia progression with less visual side effects compared with higher doses (Chia et al., 2016 ▶). The exact mechanism of non-accommodative anti-myopic activity of atropine is still unknown but there are three possible mechanisms of action (Figure 1b). It binds to the muscarinic receptors (MR) of amacrine cells in retina which in turn, increase the release of dopamine which is an inhibitory chemical mediator for eye growth. There is reduction of γ- aminobutyric acid following atropine treatment in myopia induced mice. Atropine binds to the scleral fibroblast muscarinic receptors that interfere with scleral remodelling, increase the thickness of scleral fibrous layer, reduce extracellular matrix production by decreasing glycosaminoglycan (GAG) synthesis both in the whole sclera and in isolated scleral chondrocytes (Lind et al., 1998 ▶) and upregulate the mRNA levels of MR1, MR3, and MR4 while downregulating MR2 and MR5 mRNA levels in the sclera from mouse eyes with lens-induced myopia (Barathi and Beuerman, 2011 ▶).

***Abrus precatorious*** **L.**

*Abrus precatorius* L. belong to the family of Fabaceae, its seeds are used for medicinal purposes that are hard and ovoid in shape and about 1 cm long. Chief chemical constituent is abrin which is toxic and also the herb contains several essential amino acids like alanine, serine, choline, valine, and methyl ester (Bhatia et al., 2013 ▶). It has anti-microbial, anti-inflammatory and anti-oxidant activity (Prabha et al.,2015 ▶). Umamaheswari et al (2012) ▶ investigated the anti-cataractic and anti-oxidant activity of ethanolic extract of *A. precatorius* L. seeds by calcium-induced cataractogenesis in goat lenses. Results showed significant increases in lipid hydroperoxides malondialdehyde and a decrease in protein content, Cu^2+^-induced lipoprotein diene formation and enzymatic and non-enzymatic antioxidants when compared to normal control, suggesting that *A. precatorius* L. seeds delay the progression of cataract as they protect the lens against calcium-induced oxidative damage.

***Coptis teeta*** **Wall.**

**Figure 2:**
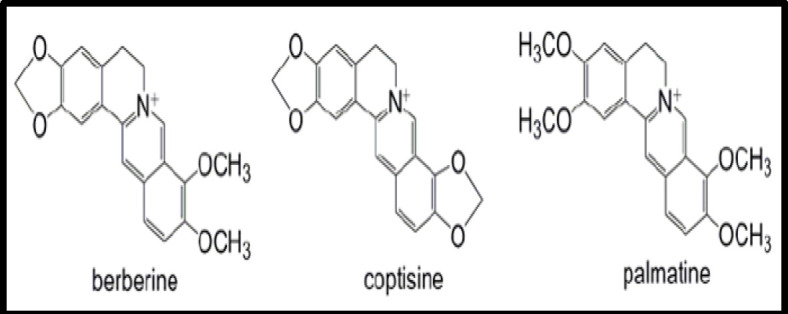
Chemical constituents of *Coptis teeta* Wall

*Coptis teeta* Wall. is a perennial herb which belongs to the family Ranunculaceae. Rhizome of this plant are used for medicinal purposes that are horizontal to oblique in shape of about 5–15 cm long with fibrous roots and bitter in taste. Externally, it is yellowish brown and pith yellow–orange and covered with numerous nodes and rootlets (Bajpay, 2019 ▶). Traditionally, it has been used in eye diseases, skin diseases, stomach problems, constipation, jaundice, urine disorders and insect bite. The active ingredient of this plant is berberine and the plant contains other alkaloids like coptisine, palmatine, epiberberine and columbamine (Figure 2). It also contains fixed oil, albumin, coloring compound, lignin and sugar (Payum, 2017 ▶). Babbar et al (1982) ▶ showed the anti-trachoma activity of the plant and concluded that berberine was more effective in eradicating Chlamydia trachoma as compared to sulfacetamide and in preventing relapse of symptoms. It also has anti-inflammatory (Jiang et al., 2015 ▶), anti-microbial, anti-bacterial (Wagner, 2004 ▶) and anti-oxidant activity (Lone et al., 2014 ▶). Abdul et al (2010) ▶ explored the anti-inflammatory and anti-histaminic activity of Unani formulation consisting of *Coptis teeta, Berberis aristate, Cassia absus, Symplocos racemose *Roxb*., *and *Azadirachta indica* in rabbits’ conjunctiva. 

***Crocus sativus*** **L** ***.***

**Figure 3:**
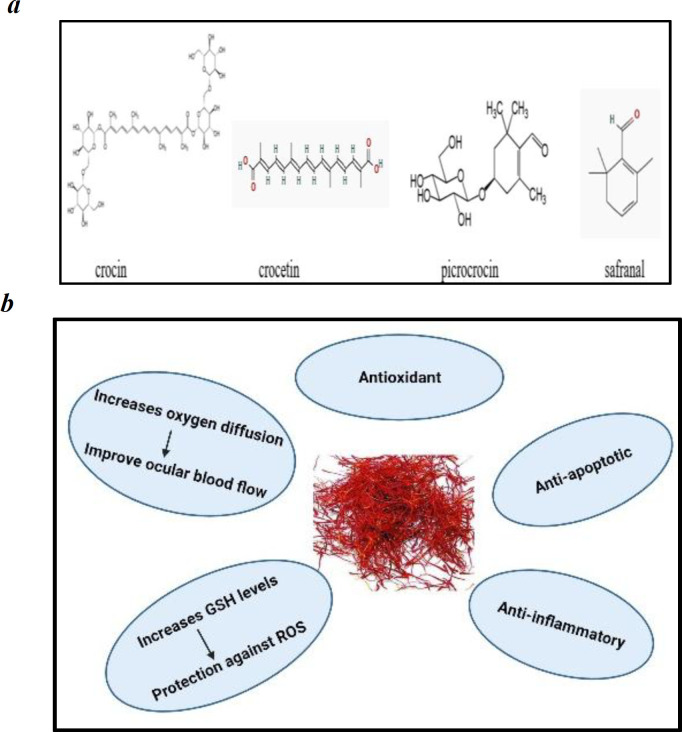
a: Chemical constituents of *Crocus sativus *L. b: Mechanism of action of *Crocus sativus* L

*Crocus sativus* L*.* is a stemless herb and it belongs to the family Iridaceae. Dried stigmas of flowers are commonly known as Saffron and used in cooking as a coloring and flavoring spice (José Bagur et al., 2017 ▶); it was known by ancient nations and has remained among the world’s costliest substances throughout the history (Mousavi and Bathaie, 2011 ▶). Phytoconstituents are crocin, crocetin, picrocrocin, and safranal as shown in Figure 3a. Traditionally, it is used as a sedative, anti- anxiety, anti-depressant, aphrodisiac, expectorant, and anti-spasmodic agent (Hosseini et al., 2018 ▶). Broadhead et al. (2019) ▶ carried out a double-blind, placebo-controlled, cross over study in age-related macular degeneration and showed that saffron increases the best corrected visual acuity. In an another double-blind, placebo-controlled clinical trial in primary open angle glaucoma (POAG) of 1-month duration, reduction of IOP was shown after three and four weeks compared to placebo, however, it returned to pre-intervention levels after a 4-week wash-out period (Bonyadi et al., 2014 ▶). In a double-masked, placebo-controlled clinical trial in diabetic macular edema, there was a significant decrease in central macular thickness and increase in best corrected visual acuity along with a significant decrease in HbA1c and fasting blood glucose (Sepahi et al., 2018 ▶). Saffron increases oxygen diffusion, improves ocular blood flow, and increases glutathione (GSH) levels that protect against reactive oxygen species (ROS) and apoptosis (Heitmar et al., 2019 ▶). The possible mechanisms of action involved in saffron effect are anti-oxidant, anti-apoptotic, and anti-inflammatory activities (Figure 3b). 

***Foeniculum vulgare*** **Mill.**

**Figure 4:**
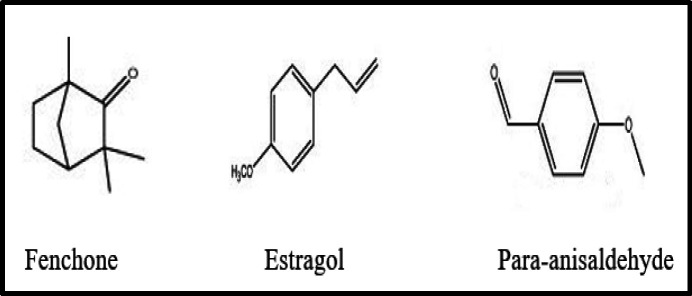
Chemical constituents of *Foeniculum vulgare *Mill

Fennel is a medicinal plant belonging to the Umbelliferae (Apiaceae) family. It has been used in traditional medicine for various ailments from thousands of years in the East Asian countries, India and China (Rather et al., 2012 ▶). The fruit and root infusions are used as relaxant, estrogenic, analgesic, and anti-inflammatory medicines. Fennel seeds have been shown to have estrogenic, antioxidant, and antihirsutism activities; it increases milk secretion, promotes menstruation, facilitates birth, and alleviates the symptoms of dysmenorrhea, and increases libido and female climacteric and essential oil has antifungal property (Mahmoudi et al., 2013 ▶). Phytochemical studies have shown the presence of numerous valuable compounds, such as volatile compounds (fenchone, estragol, and para-anisaldehyde as shown in Figure 4), flavonoids, and phenols (Miguel et al., 2010 ▶). The Romans believed that fennel seed could help supercharge the vision. It has several pharmacological properties such as anti-inflammatory, oculohypotensive, antioxidant, anti-inflammatory, antispasmodic, antiseptic, carminative, diuretic, anti-ulcer and analgesic effect (Rahimi and Ardekani, 2013 ▶). Agarwal et al. (2008) ▶ evaluated the oculohypotensive effect of aqueous extract of *Foeniculum vulgare* Mill**. **in experimental models of glaucoma. Results revealed that it exhibited 17.49, 21.16 and 22.03% reduction of intraocular pressure (IOP) in normotensive rabbits at 0.3%, 0.6% and 1.2% (w/v) concentrations, respectively. The 0.6% concentration was further evaluated in acute and chronic models of glaucoma. A maximum mean difference of 31.20% was observed between vehicle-treated and extract-treated eyes in water loading model while a maximum mean IOP lowering of 31.29% was observed in steroid-induced model of glaucoma. Mechanism of action involved in lowering IOP, might be due to its anticholinesterase activity. Another study showed the protective and therapeutic effects of aqueous extract of *Foeniculum vulgare* Mill. seed eye drops (0.5%) against selenite-induced cataract in rabbits. The results showed a highly significant reduction in lens opacity score in comparison with cataract-induced group (Hassan et al., 2017 ▶).

***Gingko biloba*** **L.**

**Figure 5:**
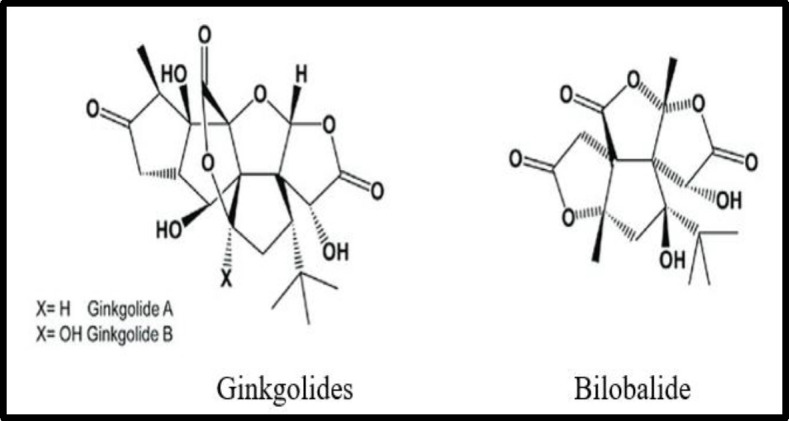
Chemical constituents of *Ginkgo biloba *L

*Ginkgo biloba* L. is one of the most popular herbal supplements used in the world. These trees are one of the oldest trees in the world and they are referred to as “living fossils” dating back over 250 million years (Niazi Mashhadi, 2021 ▶). Traditionally, this plant has been used in asthma, circulatory disorders, tinnitus, vertigo and cognitive disorders. It is often prescribed as a nootropic agent in old age and dementia. The main chemical entities are terpenoids (ginkolides, and bilobalides as shown in Figure 5) flavonoids, and proanthocyanides. It increases the ocular blood flow thereby, improves the circulation to the optic nerve head in healthy adult volunteers, and protects the retinal ganglion cells in patients with glaucoma (Park et al., 2011 ▶). *In vitro* and clinical studies suggests the use of *G. biloba* L. as an adjuvant in the treatment of normal pressure and high-pressure glaucoma that is resistant to other treatments. (Guo et al., 2014 ▶; Sari et al., 2016 ▶). 

*G. biloba* L. is considered a neuroprotective agent as it possesses antioxidant, anti-inflammatory and vasoregulatory actions and has been proposed in the treatment of glaucoma. It acts by stabilizing the mitochondria as there are abnormal mitochondrial changes that make the retinal ganglion cells more susceptible to oxidative stress (Mirzaei et al., 2017 ▶). In a study, it was found that *G. biloba* L. decreases the level of reactive oxygen species and protects the mitochondrial membrane in cultured neuronal cells (Eckert et al., 2005 ▶). It also has vasodilatory action which helps in improving the coronary and peripheral circulation and blood viscosity. It lowers the low-grade inflammation by reduction in active cells (e.g., glial cells) in low grade inflammation (Ige and Liu, 2020 ▶). Randomized clinical trials showed improvements in visual field (VF) indices in normal tension glaucoma (NTG) with significant increases in ocular blood flow, blood volume, and velocity (Shim et al., 2012 ▶). Another study showed a significant improvement in VF indices, superior and inferior retinal nerve fiber layer thickness, malondialdehyde (a plasma derived oxidative stress marker), and glutathione peroxidase (an antioxidant enzyme) in primary open angle glaucoma (POAG), but no significant changes in IOP (Sari et al., 2016 ▶). 

***Zingiber officinale*** **Roscoe**

**Figure 6:**
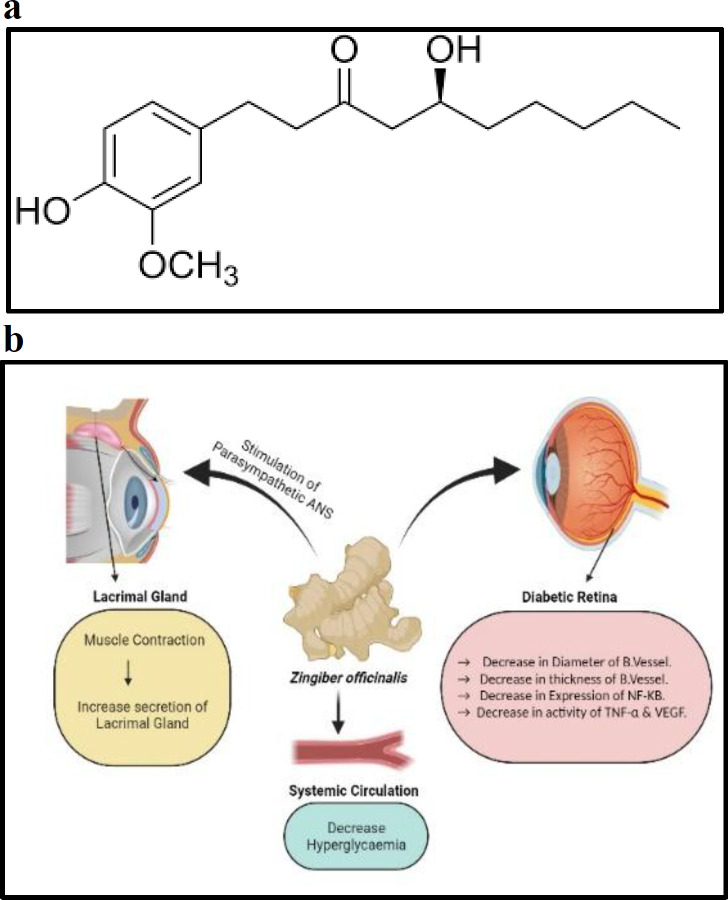
a: Chemical structure of *Gingerol* b: Mechanism of action of *Zingiber officinalis*

*Zingiber officinale* Roscoe is a perennial herb with fleshy underground rhizomes. It is edible and it has high nutritional and medical values. Various scientific studies reported anticancer, antihypercholesterolaemic, antiarthritis, antibacterial, antiviral, fever reducing, antimetic, and antiulcer effects. The main active component is gingerol as shown in Figure 6a (Dhanik et al., 2017 ▶). Dongare et al., (2016) ▶ investigated the retinal microvascular changes in diabetic rats treated with *Zingiber officinale* Roscoe extract containing 5% of 6-gingerol, orally. Results showed a significant reduction in hyperglycemia, the diameter of the retinal vessels, and vascular basement membrane thickness. Improvement in the structural changes of the retinal vasculature was associated with significantly reduced expression of nuclear factor-κB (NF-κB) and reduced activity of tumour necrosis factor-α (TNF-α) and vascular endothelial growth factor (VEGF) in the retinal tissue. In another study, the anti-glaucoma activity of aqueous methanolic extract of ginger against carbomer-induced experimental glaucoma in rabbits have been evaluated (Pathan and Ali, 2014 ▶). Results showed a significant decrease in IOP and serum pseudocholinesterase (Figure 6b). Another study revealed that consumption of (4 g) of fresh ginger has a significant effect on the rate of tear production. Gingerol has a stimulatory action on parasympathetic autonomic nervous system (ANS) that results in contraction of muscles and increases in glandular secretion (lacrimal gland) (Uzodike and Ilodibe, 2010 ▶.). Akbari et al. (2020) ▶ showed that ginger extract has protective effects against toxicity induced by ethanol in the eye of male rats, by its antioxidant activity and through improving regulation of homocysteine synthesis and homeostasis of trace elements.

## Discussion

Since the use of physostigmine as the first herbal product having an application in ophthalmology, nature remains an important source of substances with proven benefits to ocular health. However, while there has been an exponential increase in the number of natural compounds reputed to have ocular activity, most have not been sufficiently studied to prove their safety and efficacy. Therefore, scientific support is lacking, despite the promotion of a growing number of such compounds, including those for which patents are pending. On the other hand, continued research may enhance the ability to synthesize new compounds based on nature-derived prototypes, giving rise to new pharmacologic options with additional properties. In addition, already-established molecules may be refined to optimize their pharmacokinetics or improve drug delivery systems. Despite recent progression in ophthalmic medicine, still there are several refractory eye diseases that require special attention to develop un- explored fields of medical knowledge. There are several traditional medicinal plants that are in ophthalmic practices and are evident for their safety and efficacy but they lack scientific validation. A special attention should be given to analyze these drugs and their pharmaco-dynamic and kinetic principles should be thoroughly investigated. 

The use of medicinal plants in modern medicine suffers from the fact that although hundreds of plants are used in the world to prevent or to cure diseases, scientific evidence in terms of modern medicine is lacking in most cases. However, today it is necessary to provide scientific proof as to whether or not it is justified to use a plant or its active principles.

## Conflicts of interest

The authors have declared that there is no conflict of interest.
